# *Crotalus durissus collilineatus* Venom Induces TNF-****α**** and IL-10 Production in Human Peripheral Blood Mononuclear Cells

**DOI:** 10.1155/2014/563628

**Published:** 2014-01-19

**Authors:** Camila Bastos Ribeiro, Jéssica Cristina dos Santos, Jacyelle Medeiros Silva, Pedro Henrique Silva de Godoi, Marta Regina Magalhães, Mônica Spadafora-Ferreira, Simone Gonçalves Fonseca, Irmtraut Araci Hoffman Pfrimer

**Affiliations:** ^1^Departamento de Biomedicina e Farmácia, Pontifícia Universidade Católica de Goiás, 74605-140 Goiania, GO, Brazil; ^2^Centro de Estudos e Pesquisas Biológicas (CEPB), Pontifícia Universidade Católica de Goiás, 74605-010 Goiania, GO, Brazil; ^3^Laboratório de Imunogenética, Instituto Butantan, 05503-900 São Paulo, SP, Brazil; ^4^Instituto de Patologia Tropical e Saúde Pública, Universidade Federal de Goiás, 74605-050 Goiania, GO, Brazil

## Abstract

Snake venom has been the subject of numerous studies in an attempt to find properties and biological effects that may be beneficial to man. In this study we evaluated *in vitro* the effects of *Crotalus durissus terrificus* (Cdt) and * Crotalus durissus collilineatus *(Cdc) venom in human peripheral blood mononuclear cells (PBMCs). At 24 h, a significant decrease of viable cells was observed in cells stimulated with the Cdc venom at 0.0005 mg/mL and 0.005 mg/mL compared to the negative control. At 48 h, a significant decrease of viable cells was observed only in cells stimulated with Cdc venom at 0.005 mg/mL. A significant increase of TNF-**α** and IL-10 was detected 48 hours after culture of PBMC with Cdc, but not with Cdt venom. The expression of CD69 and PD1 (programmed death-1), activation and regulatory cell markers, on CD8+ and CD8− T cells did not change in the presence of Cdt and Cdc venom. Our results suggest the presence of proinflammatory and anti-inflammatory components in the Cdc venom. Further analysis should be done to identify those Cdc venom components as it has been done for the Cdt venom by other authors, indicating that modulatory components are found in the venom of different species of *Crotalus* snakes.

## 1. Introduction

Snakes are included in one of the most diverse groups of reptiles in the world, with over 3100 known living species [1]. Brazil has cataloged 721 species of snakes. Only two families (Elapidae and Viperidae) show venomous species, those that produce toxins in specialized glands [2].

Snakes belonging to Viperidae family and *Crotalus* genus are represented in Brazil by a single species, *Crotalus durissus* and five subspecies known as *Crotalus durissus terrificus* (Cdt), *Crotalus durissus collilineatus *(Cdc), *Crotalus durissus cascavella*, *Crotalus durissus ruruima,* and *Crotalus durissus marajoensis*. The genus *Crotalus*, which is popularly known as rattlesnakes, whose venom is the subject of this study, has been highlighted in the research and its venom has the highest lethality rate [3–5].

Snake venom is a complex mixture consisting of proteins, peptides, amino acids, nucleotides, lipids, and carbohydrates that have a wide variety of biological activities that reflect changes in the biological properties and toxicity [6, 7].

The venom of the rattlesnakes is considered one of the most toxic venom from Brazilian snakes. The high toxicity is due to the presence of various toxins [8], mainly convulxin, crotamine, crotoxin, and gyroxin [9]. Despite all the toxic effects, the venom usually is a rich source of molecules with important pharmacological properties that have great potential for the production of new drugs [10–12].

It has been shown that snake venom can induce inflammatory cytokine production and cell death [13]. In the present work, we investigate the cytotoxicity, the cytokine production, and the modulation of activation or regulation markers in human peripheral blood mononuclear cells treated with two concentrations of *Crotalus durissus terrificus* and *Crotalus durissus collilineatus* venom. The results show a decrease of cell viability and increase of TNF-*α* and IL-10 production in presence of Cdc venom, suggesting the presence of proinflammatory and anti-inflammatory fractions on the crude venom.

## 2. Materials and Methods

### 2.1. PBMCs Isolation

Human PBMCs were isolated from heparinized blood of 12 healthy individuals, aged between 18 and 40 years. The heparinized whole blood was centrifuged for 20 minutes at 2000 rpm at a temperature of 18°C to obtain leukocyte ring. The leukocyte ring was extracted, transferred to a Falcon tube, and added to phosphate buffered saline (PBS) to complete 10 mL. Subsequently, to obtain the PBMC, the homogenate was applied on the medium density gradient (Ficoll-paque density = 1.077 g/L, Amersham). This solution was centrifuged for 20 minutes at 950 g at a temperature of 18°C to obtain leukocyte cloud. The cells from the interphase were extracted with the aid of a Pasteur pipette and washed 3 times in PBS (10 minutes, 800 g, 18°C). After the last wash, cells were maintained in RPMI 1640 medium with 20 mM Hepes supplemented with 10% Fetal Bovine Serum (FBS), 2 mM L-glutamine (Gibco/Invitrogen, Grand Island, NY).

### 2.2. Venom

Venom of *Crotalus durissus terrificus* and *Crotalus durissus collilineatus* venom was extracted by manual massage of the venom gland, and after that they were clarified by centrifugation at 4000 g for 15 minutes at 4°C and lyophilized. These Cdt and Cdc venom were obtained from Centro de Estudos e Pesquisas Biológicas (CEPB), Pontifícia Universidade Católica de Goiás (PUC-GO), Goiania, Brazil, and stored at −20°C. The venom was dissolved in PBS, immediately before use. Two concentrations of the venom were previously tested and found to be cytotoxic at 0.005 *μ*g/mL and noncytotoxic at 0.0005 *μ*g/mL of Cdt and Cdc (unpublished data) and were chosen.

### 2.3. Cell Viability

Cell viability was used to determine total cell counting in Neubauer chamber after dilution in Trypan blue (1 : 2, v/v) (Gibco: 0.4%).

### 2.4. Cell Culture

Fresh PBMCs from healthy donors were cultured in 96-well plates at concentration of 2 × 10^5^ cells/mL in absence or presence of 0.005 *μ*g/mL or 0.0005 *μ*g/mL of Cdt or Cdc venom for 24, 48, and 72 hours. The cell viability was determined at 24, 48, and 72 h. Expression of CD69 and PD1 on T cells were analyzed 24 h after venom stimulation. Culture supernatants were harvested 48 h after stimulation and tested for the presence of cytokines.

### 2.5. Cytokine Detection

Cytokines in culture supernatants were quantified by flow cytometry (FACSCalibur flow cytometer, Becton Dickinson, California, USA) using Human Th1/Th2 Cytokine Cytometric Bead Array (CBA) reagent kit (BD Biosciences Pharmingen, California, USA) according to the manufacturer's instructions. The production of IL-2, IL-4, IL-6, IL-10, TNF-*α*, and IFN-*γ* cytokines was quantified. The results were based on a standard concentration curve established between 5.000 and 20 pg/mL.

### 2.6. Flow Cytometry Analysis

After 24 h of Cdt and Cdc venom stimulation, PBMCs were collected and stained with antihuman PD1 PE, antihuman CD8 PerCP-Cy5.5, and antihuman CD69 APC-Cy7 (FN50) (all antibodies were from BD Pharmingen, San Diego, CA) for 30 minutes at 4°C. Cells were washed twice with PBS, resuspended in 1% paraformaldehyde, and analyzed. A total of 10.000 events were acquired per sample using an FACS Canto (Becton Dickinson, San Jose, CA) and the analyses were done using FlowJo software (Tree Star).

### 2.7. Statistical Analysis

The data were expressed as median of tested individual for each condition. Statistical significance was determined using a nonparametric test, Mann-Whitney, among control and cells stimulated with Cdt and Cdt venom (0.005 *μ*g/mL and 0.0005 *μ*g/mL); *P* < 0.05 was considered significant.

### 2.8. Ethics Statement

The written informed consent from blood donors with respect to taking blood samples for research purposes was obtained and approved by the Ethics in Research of the Federal University of Sao Paulo (UNIFESP) no. 0308/10.

## 3. Results

### 3.1. Evaluation of PBMC Viability

**Figure 1:**
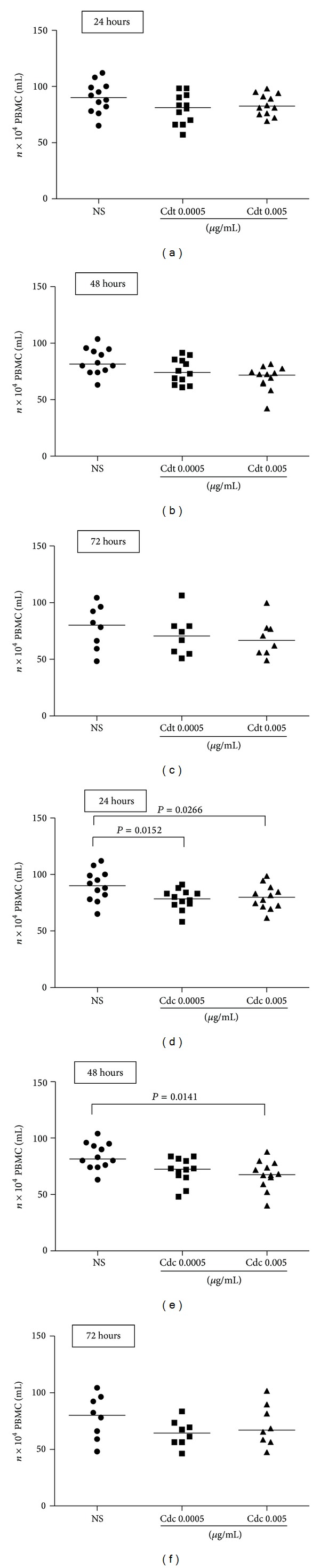
PBMC counts after 24, 48, and 72 hours of culture in the presence and absence of Cdt and Cdc venom. An initial amount of 2 × 10^5^ cells/mL was stimulated with Cdt venom and Cdc venom in concentrations of 0.0005 *μ*g/mL and 0.005 *μ*g/mL and only with RPMI (NS). (a) PBMC counts in 24 hours with Cdt venom; (b) PBMC counts in 48 hours with Cdt venom; (c) PBMC counts in 72 hours with Cdt venom. (d) PBMC counts in 24 hours with Cdc venom; (e) PBMC counts in 48 hours with Cdc venom; (f) PBMC counts in 72 hours with Cdc venom. The analysis was done by Mann-Whitney test and the horizontal bars show the median.*P < 0.05 when compared with the corresponding negative control. Cdt: *Crotalus durissus terrificus *venom; Cdc: *Crotalus durissus collilineatus *venom.

To assess whether the Cdt and Cdc venom was able to induce changes on cell viability, human PBMCs were cultured in the presence of those two concentrations of venom and cell viability was evaluated 24, 48, and 72 h after venom stimulation and the results are shown in Figure 1. A significant decrease of viable cells was observed at 24 h in the condition of cells stimulated with the Cdc venom at concentrations of 0.0005 *μ*g/mL and 0.005 *μ*g/mL compared to the condition of nonstimulated cells (negative control) (Figure 1(d)). After 48 hours of venom stimulation a significant decrease of viable cells was observed only between cells stimulated with the Cdc venom at concentration of 0.005 *μ*g/mL compared to the negative control (Figure 1(e)). There were no changes in the cell viabiltiy when PBMC were stimulated with Cdt venom for 24 h (Figure 1(a)) and 48 h (Figure 1(b)). After 72 h of stimulus there was no significant difference observed between the tested conditions (Figures 1(c) and 1(f)).

### 3.2. Cytokine Production upon Stimulation of PBMC with Cdt and Cdc Venom

**Figure 2:**
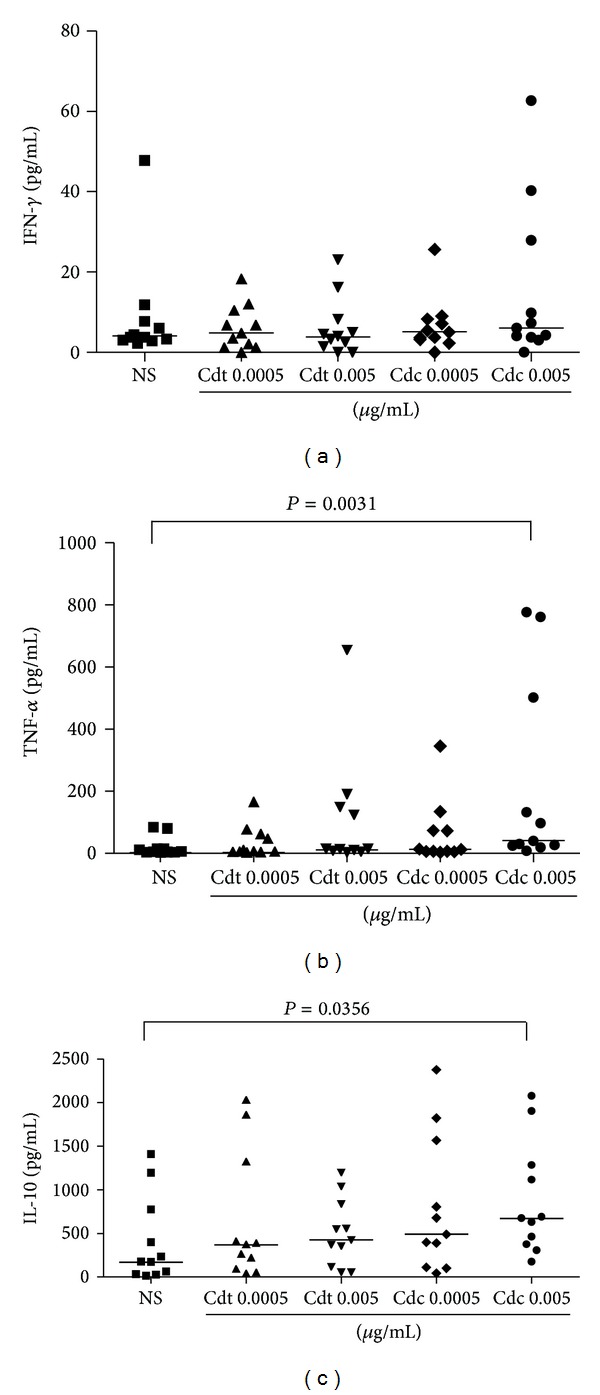
Concentration of cytokines produced by PBMCs after 48 hours of culture in the presence and absence of Cdt and Cdc venom. Concentrations of (a) IFN-*γ*, (b) TNF-*α*, and (c) IL-10. 2 × 10^5^ cells/mL were stimulated with different concentrations of the venom and the culture supernatants were collected after 48 hours. Cytokines in the supernatants were measured by Cytometric Bead Array. The analysis was done by Mann-Whitney test and the horizontal bars show the median.*P < 0.05 when compared with the corresponding negative control. Cdt: *Crotalus durissus terrificus* venom; Cdc: *Crotalus durissus collilineatus *venom; NS: nonstimulated cells.

In order to investigate the effect of Cdt and Cdc venom on cytokine production we cultured PBMC with the venom at concentrations of 0.005 *μ*g/mL and 0.0005 *μ*g/mL. In the evaluation of cytokine production by PBMC in 48 hours, we observed a significant increase of TNF-*α* in cells exposed to Cdc venom at concentration of 0.005 *μ*g/mL (Figure 2(b)) and increase of IL-10 in cells exposed to Cdc venom at concentration of 0.005 *μ*g/mL (Figure 2(c)). The same increase was not observed for IFN-*γ* (Figure 2(a)), IL-2, and IL-4 (data not shown).

### 3.3. Evaluation of Activation and Regulation Markers on Cells Stimulated with Crotalus Venom

**Figure 3:**
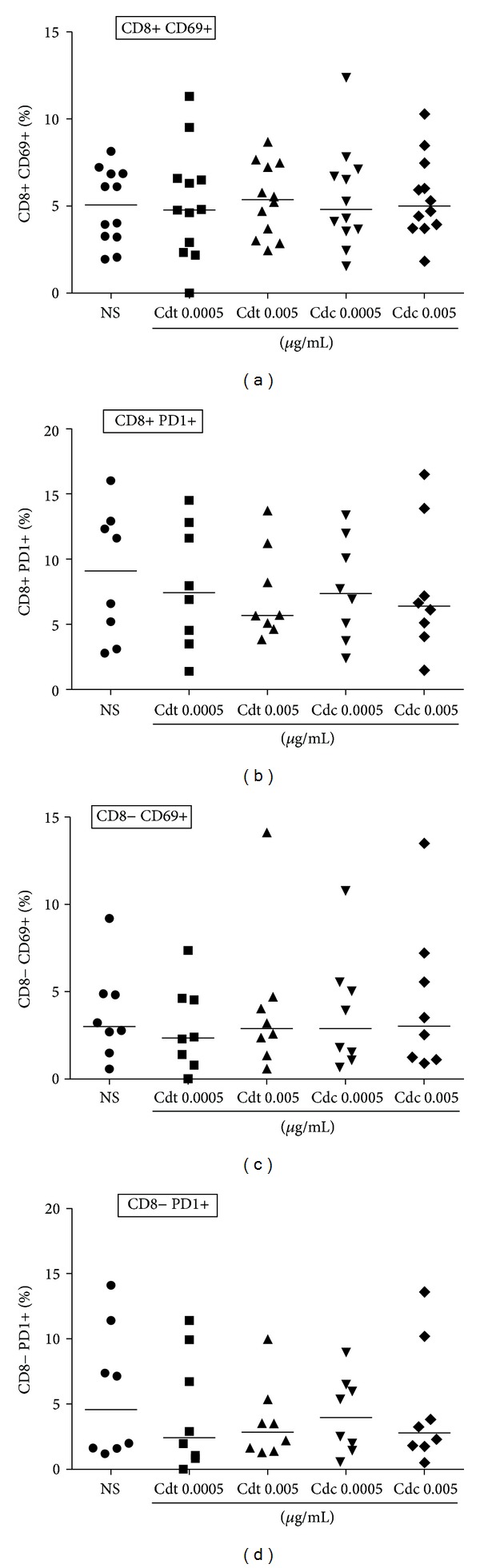
Expression of activation (CD69) and regulation (PD1) markers. PBMCs were cultured in presence of Cdt and Cdc venom. After 24 h, cells were stained with antibodies for CD8, PD1, and CD69 and analyzed by flow cytometry. A gate was done in the region of lymphocytes and those cells were gated as CD8+ and CD8− subsets and the expression of PD1 and CD69 was analyzed inside of these subsets. (a) CD69 expression on CD8+ T cells. (b) PD1 expression on CD8+ T cells. (c) CD69 expression on CD8− T cells. (d) PD1 expression on CD8− T cells. The analysis was done using Mann-Whitney test and the horizontal bars show the median. Cdt: *Crotalus durissus terrificus* venom; Cdc: *Crotalus durissus collilineatus *venom; NS: nonstimulated cells.

Given the polarized pattern of cytokine production by PBMC cultured in presence of Cdc venom, we aimed to investigate whether the venom would induce an early activation or regulation of T cells that could be associated with the production of TNF-*α* and IL-10, respectively. For that, we investigated the expression of CD69, a marker of early activation and programmed death 1 (PD1), an inhibitory marker, on PBMC cultured in presence of Cdc and Cdt. In the population of CD8+ T cells a slight trend of increasing CD69 expression was observed after culture with both Cdc and Cdt venom (Figure 3(a)), although, not significant. There was no significant difference in the expression of PD1 on CD8+ T cells (Figure 3(b)). In the population of CD8− T cells (mainly CD4+ T cells) there were no significant differences in the expression of CD69 and PD1 (Figures 3(c) and 3(d)).

## 4. Discussion

There is a high interest in identifying molecules in snake venom with important pharmacological properties for development of new drugs. Several studies have been done with the aim of elucidating the effects of the venom toxins in an attempt to find substances that can bring some benefit to man. Studying cell behavior in face of toxins is very important to understand the mechanisms involved in the tissues when venom infiltrates the organism in a snake bite, so *in vitro* tests should be performed first [14]. In this study, we investigated the effect of Cdt and Cdc venom on the viability and cytokine production of human peripheral blood mononuclear cells. Our results showed that Cdc venom induced decrease in the cell numbers and induced TNF-*α* and IL-10 production by human PBMC. Our results suggest that the Cdc crude venom may contain distinct components that stimulate proinflammatory cytokines as TNF-*α* and anti-inflammatory cytokines as IL-10.

Previous studies from our group were performed in order to identify the concentration of the venom with lower cytotoxic effect on PBMC. These results showed that the concentration of the Cdt and Cdc venom that caused no cytotoxicity in PBMC was 0.0005 *μ*g/mL (unpublished data). According to this data we tested venom at 0.0005 *μ*g/mL as a noncytotoxic concentration and 0.005 *μ*g/mL as a test dose. First, we analyzed the effect of the venom at those concentrations on PBMC to exclude the cytotoxic effects and to investigate the potential to induce cell proliferation of the venom. We did not detect proliferative effect on both venom, but we could observe some decrease of cell numbers, suggesting a cytotoxic effect in the higher dose of the venom. However, in this study we did not investigate the apoptosis markers such as annexin V and caspases to clarify the decrease of cell viability. In this direction, it has been shown that crotoxin, the main component of Cdt venom, has been characterized as an immunomodulatory molecule both *in vitro* [15] and *in vivo* [16]. Alterations in leukocyte distribution characterized by a drop in the number of monocytes and lymphocytes and an increase in neutrophils with an increase in serum IL-6 and IL-10 were observed in mice after subcutaneous injection of crotoxin [15]. Later, the same group showed that the venom as well as crotoxin has inhibitory effect on mice splenic cells proliferation with no decrease in cell viability. In addition, they showed that the venom diminished the levels of IL-2, IL-4, IL-10, and IFN*γ* [17]. Moreover, it was also shown that crotoxin inoculation is also able to suppress antibody production in human serum albumin immunized mice [16].

To further analyze and compare the effect of the Cdt and Cdc venom on functional capacity of the PBMC we cultured those cells with different concentrations of the venom and analyzed the cytokine production in the culture supernatants. We tested Th1 and Th2 cytokine patterns including IL-2, IL-4, IL-10, TNF-*α*, and IFN-*γ*. TNF-*α* is a proinflammatory cytokine involved in several processes such as inflammation and control of infectious diseases [18]. The present study shows an increase of TNF-*α* and IL-10 at 48 hours after Cdc venom stimulation at concentration of 0.005 *μ*g/mL but not at lower concentration (0.0005 *μ*/mL). Our data did not show changes at the levels of IL-2, IL-4, and IFN-*γ* in the supernatants of the cell cultures treated with the same venom at 48 hours. However, we cannot exclude the possibility that IL-10 could have modulate IL-2 and IFN-*γ* production, since the main effect of IL-10 is to inhibit the synthesis of other cytokines, such as IFN-*γ*, IL-2, IL-12, and TNF-*β* [19].

In order to investigate some possible mechanisms involved in the increase of TNF-*α* and IL-10, we investigate the expression of CD69 as an early activation cell marker and PD1 as a regulatory cell marker. CD69 is a very early activation marker on T lymphocytes [20]. A recent work showed that disintegrins, a family of polypeptides released in the venom snakes that selectively block the function of *β*-integrin, were able to induce *in vitro* T-cell proliferation and increase of CD69 expression on human T lymphocytes [21]. Programmed death 1 (PD1) is a coinhibitory receptor and its expression can be induced primarily on the cell surface of activated CD4+ and CD8+ T cells. The interaction of PD1 and its ligand cause inhibitory effects in the antigenic stimulation [22]. It has been shown that the triggering of PD1 in monocytes can induce the IL-10 production and inhibits the CD4 T-cell function [23]. We did not find modulation in the expression of CD69 and PD1 molecules on CD8+ and CD8− T cells at 24 h of stimulation with Cdt or Cdc venom at the tested concentration. Our data suggest that in these conditions the T lymphocytes may not be the major cell population responsible for the production of TNF-*α* and IL-10 after Cdc venom stimulation.

Our study shows that the elevation of concentrations of TNF-*α*, which occurs 48 hours after cell culture, was induced by Cdc venom and no significant changes of this cytokine was observed with Cdt venom. In a recent study, BALB/c female mice were injected with 10 *μ*g/g Cdt venom and the concentration of cytokines in serum was determined after bleeding at different time intervals. The results showed that venom of Cdt induced a discrete increment of IL-6 levels at 15 minutes after injection. The TNF-*α* and IFN-*γ* levels increased gradually, reaching their highest levels at 2 hours after injection, decaying thereafter. Cdt venom was also capable to induce an increase in the serum levels of IL-4 and IL-10 with the highest values occurring 4 hours after injection, decaying thereafter [14]. The same group has shown that Cdt venom induces, *in vitro*, the inflammatory cytokines TNF-*α* and IL-6 but did not alter IL-10 production by murine macrophages [6]. The discrepancy in those results could be due to many factors, including the differences in the methods of venom extraction, the concentrations of the venom, the *in vitro* and *in vivo* models, and the mice and human systems.

IL-10 is an anti-inflammatory and immunoregulatory cytokine due to its capacity of inhibiting the proinflammatory cytokine secretion [24] and regulating the differentiation and proliferation of many cells [25]. The present study shows that the levels of IL-10 increased after 48 h of cell culture with Cdc venom at the concentration of 0.005 *μ*g/mL. The concomitant presence of TNF-*α*, a proinflammatory cytokine, and IL-10, anti-inflammatory cytokine, in the supernatants of cell culture stimulated with the Cdc venom is intriguing. It may suggest the involvement of distinct cell subsets, either lymphocytes or monocytes, being stimulated by different fractions of Cdc venom. This is the first *in vitro* study showing the effect of *Crotalus durissus collilineatus* venom on human-derived immune cells. All data from the literature are from the venom of *Crotalus durissus terrificus.* Further investigations should be done to identify if the same components that are present in Cdt venom like crotoxin are responsible for the two patterns of cytokine production induced by Cdc venom. This will open the possibilities to use these fractions as modulators of the immune response.
